# Exploring the impact of age, sex and life experiences on plasma inflammatory profiles through comparative proteomics

**DOI:** 10.3389/fimmu.2025.1695213

**Published:** 2026-01-07

**Authors:** Martina Bartolucci, Olga Utyro, Anita Muraglia, Alessia Repetto, Vanessa Agostini, Monica Pizzonia, Silvia Ottaviani, Gino Tripodi, Gilberto Filaci, Ranieri Cancedda, Andrea Petretto, Maddalena Mastrogiacomo

**Affiliations:** 1 Core Facility for Omics Sciences, IRCCS Istituto Giannina Gaslini, Genoa, Italy; 2 Department of Internal Medicine and Medical Specialties (DIMI), University of Genoa, Genoa, Italy; 3 Transfusion Medicine Department, IRCCS Ospedale Policlinico San Martino, Genoa, Italy; 4 Geriatric Clinic, IRCCS Ospedale Policlinico San Martino, Genoa, Italy; 5 Immunohematology and Transfusion Medicine Unit, IRCCS Istituto Giannina Gaslini, Genoa, Italy; 6 Biotherapies Unit, IRCCS Ospedale Policlinico San Martino, Genoa, Italy; 7 University of Genoa, Genoa, Italy

**Keywords:** plasma, proteomics, mass spectrometry, aging, inflammation, immunity, senescence

## Abstract

**Background:**

In the heterochronic parabiosis model it has been shown that blood from elderly animals exhibits markedly reduced rejuvenating effects compared to that of young organisms. Furthermore, human plasma from older subjects, when used as a supplement in cell culture media, is significantly less effective than plasma derived from younger individuals. This study analyzed plasma from a cohort of 229 subjects by a proteomic approach to reveal age-related changes.

**Methods:**

A mass spectrometry-based proteomic analysis was performed on plasma samples from 3 age-groups: a prepubertal, a healthy young adult group and a cohort of individuals over 75 years old with three different life-experiences. An additional parallel study was conducted by a Milliplex Luminex assay.

**Results:**

The proteomic analysis revealed a chronic inflammatory state in the elderly population, along with complement activation and impaired regulation of blood coagulation. This inflammatory condition was confirmed by Luminex assay, showing elevated levels of classical pro-inflammatory cytokines in the plasma of elderly individuals. Moreover, the elderly group showed a reduced production of antibody light chains, suggesting concurrent immunosenescence. In the older group we identified 25 upregulated proteins whose elevated abundance, combined with acquired immune aging may constitute a plasma proteomic signature of aging. The degree of upregulation of these signature proteins varied among elderly subgroups with different life-experience. A good physical condition and/or cognitive function correlated with a lower expression of the aging-related proteomic profile. Furthermore, several sex-specific differences were identified in the plasma profiles of young donors. Reversely, among elderly individuals, no major differences were observed, except for an increased level of Pregnancy Zone Protein (PZP) in females.

**Conclusions:**

Proteomic analysis of plasma revealed protein variations associated with aging, primarily involving inflammation-related pathways, immunosenescence features, and sex-linked differences. This study highlights the pathological characteristics underlying the aging process.

## 1 Introduction

Aging is linked to a progressive loss of homeostasis and regenerative potential of the organism. Some studies showed an “aging” effect of exposing young tissues to an old environment. When mice of different ages were joined by heterochronic parabiosis, in response to the young organism, the old animal showed rejuvenation of aged progenitor cells and higher tissue regeneration properties (1). On the contrary, young animals exposed to an old systemic environment or to plasma infusion from an old organism showed decline in neurogenesis and cognitive impairment (2). Differences in the nature and concentrations of systemic regulators of tissue physiology present in the blood may be responsible of the observed effects in the parabiotic experiments. “Rejuvenating” effects were also obtained by the intravenously injection of young plasma in old mice (3). This raised hopes of slowing down the senescence process in humans by a similar approach. Some clinical studies have been performed (4, 5), however, solid evidence is still missing to support the “rejuvenating” effect of young plasma injection in humans.

Human plasma and serum have been proposed as culture medium supplements to replace fetal calf serum. We recently reported that plasma derived from old subjects was less efficacious in supporting cell proliferation than the one obtained from young blood donors (6). The reduced cell growth induction could be due to inhibitors present in the old plasmas and/or to the missing or decreased concentration of specific activators present in the young plasmas. We have performed an initial study of the different protein content of young and old plasmas using a commercially available cytokine array for parallel determination of the relative levels of 105 selected human proteins. Specific differences between young and old plasmas were revealed with a higher concentration of “inflammatory” proteins in old ones (6). However, our previous study was limited to few plasma samples and to the analysis of few tens of proteins. Based on these observations, our aim was to investigate whether differences in the protein content of plasmas derived from young and old individuals could correlate with differences in their regeneration induction potential, and to better understand the molecular basis underlying age-related functional decline. Only a proteomics approach on many samples could provide complete and unbiased results of the different protein content of young and old plasmas.

So far, numerous studies have investigated the differences in plasma protein composition between young and elderly individuals. The sample size in these studies have ranged from a few dozen participants (7, 8) to several thousand (9–11), with one notable study analyzing plasma from over fifty thousand individuals (12). Donor age has ranged from newborns to centenarians. Despite some variability in the depth of information provided, these studies consistently revealed significant age-related differences in both the nature and concentration of circulating plasma proteins. Among the proteins upregulated in elderly individuals, some - particularly those associated with inflammation - were frequently identified across multiple studies. However, many publications primarily present lists of proteins elevated in aging plasma, often without exploring their biological functions or the signaling pathways they influence. In several cases, studies have focused on individuals with specific diseases (e.g., cancer) versus healthy controls or have investigated genetic variations in plasma proteins as predictors of disease risk in otherwise healthy populations. Few, if any, studies have systematically examined subgroups of elderly individuals with varying lifestyle factors (13, 14), leaving a gap in our understanding of how life-experience modulates the plasma proteome in aging.

Here, we present proteomic analysis of plasma from individuals over 75 years of age with different life-experience and compared them to those from young adults and prepubertal children. Our findings suggest that physiological aging - combined with lifelong exposure to micro- and macro- traumatic events - initiates a cascade of specific biological processes. These include the secretion of Senescence-Associated Secretory Phenotype (SASP) factors, the release of Damage-Associated Molecular Patterns (DAMPs), changes in immune complex dynamics, complement system activation, and dysregulation of coagulation pathways. Collectively, these processes contribute to the establishment of a chronic inflammatory state in the elderly.

Furthermore, alterations in the composition and concentration of immunoglobulin chains observed in elderly plasma are indicative of an immunosenescence state. Importantly, our analysis revealed significant differences in plasma protein profiles among elderly subgroups characterized by distinct life-experience, highlighting their potential modulatory role in the aging-associated plasma proteome. Among elderly individuals, the most notable finding was an elevated level only of Pregnancy Zone Protein (PZP) in females, while in young subjects more sex-dependent differences were observed.

## 2 Materials and methods

### 2.1 Plasma samples

Blood was obtained from three groups of human donors (**Table 1**): 102 subjects older than 75 years, 107 blood donors aged 18 to 25 and 20 prepuberal children. The more than 75 elderly group included 3 sub-groups: O-ct) 26 volunteer donors in “relatively” good health. This group included several senior members of a hiking club performing one day mountain hike at least weekly and several university emeritus professors still intellectually active; O-g) 32 patients of the geriatric clinic. This group included several patients with cognitive deficit; O-o) 44 individuals which were hospitalized for a bone fracture. After hospitalization, in almost all patients were diagnosed mild or moderate traits of cognitive deficit. From these patients, plasma was collected usually 1–2 days after fracture.

**Table 1:** Blood donor group characterization.

Name	n	Age range, y	Age mean, y	Male	Female
**Old**	**102**	**75-100**	**84.3**	**41**	**61**
Old-ct	26	75-96	79	20	6
Old-g	32	75-93	83.9	15	17
Old-o	44	75-100	87.6	6	38
**Young**	**107**	**18-25**	**22.1**	**52**	**55**
**Child**	**20**	**1-11**	**6.8**	**12**	**8**

Bold values represent the main group numbers.

For plasma preparation, whole blood, harvested into K3-EDTA containing sampling tubes, was centrifuged at 1,600 g for 15 min at 4°C. The supernatant, corresponding to the plasma fraction was recovered, divided into aliquots, and stored at -80 °C for further use.

### 2.2 Mass spectrometry

For the mass spectrometry analysis freeze-stored plasmas were thawed, 5 μl of plasma was lysed, reduced and alkylated in 60 μl LYSE buffer (Preomics, Martinsried, Germany) for 10 min at 95 °C and 1,000 rpm mixing. From each sample 50 µg of proteins were digested by PAC method automated on a KingFisher™ Apex robot (Thermo Fisher Scientific, Waltham, MA, USA) in 96-well format as described in (15). The tip plate was stored in plate #1. Lysate samples were stored in plate #2, at a concentration of 70% acetonitrile and with magnetic beads in a protein/bead ratio of 1:4 (1:1 SpeedBead Magnetic Carboxylate, 45152105050250 and 65152105050250). Washing solutions were in plates #3–5 (acetonitrile), plate #6 (70% Ethanol) and plate #7 (isopropanol). Plate #8 contained 100 ml digestion solution of 25 mM Tris HCl pH 8, LysC (Wako, Osaka, Japan) in an enzyme/protein ratio of 1:100 (w/w) and trypsin (Promega, WI, USA) in an enzyme: protein ratio of 1:50. The protein aggregation was carried out in two steps of 1 min by mixing at medium speed, each step followed by a 10 min pause. The sequential washes were performed in 2.5 min at slow speed, without releasing the beads from the magnet. Digestion was for 4 h at 37 °C at slow speed. Obtained peptides were analyzed on the Evosep One system using an Endurance column (8 cm x 100 μm, 3 μm particle size, Evosep) and the pre-programmed gradient of 60 sample per day, with a flow rate of 1 ml/min. The column was maintained at room temperature and interfaced online with the Orbitrap Exploris 480 MS (Thermo Scientific, Odense, Denmark) with FAIMS Pro Duo Interface (Thermo Scientific). MS analysis was performed in DIA mode. FAIMS CV was set to -45 at standard resolution. Full MS resolution was set to 60,000 in a range between 380 and 980 m/z and with a normalized AGC target of 300% with a maximum IT set to Auto. Normalized AGC target value for fragment spectra was set at 1,000%. 50 windows of 12 Da were used with an overlap of 1 Da. Resolution was set to 15000 and IT to Auto. HCD collision energy was set at 25%. All data were acquired in profile mode using positive polarity.

All DIA raw files were processed with Spectronaut version 18 using a library-free approach (directDIA) under default settings (16). Library was generated against UniProt Human database (release UP000005640_9606 November 2022, 102572 entries). Carbamidomethylation was selected as a fixed modification, while methionine oxidation, N-terminal acetylation, and Deamidation (NQ) were selected as variable modifications. FDRs of PSMs and peptide/protein groups were set to 0.01. For quantification Precursor Filtering was set to Identified (Q value) and MS2 was chosen as quantity MS-level.

### 2.3 Protein detection by Milliplex Luminex

Expression of GM-CSF, IFNG (IFNγ), IL1A, IL1B, IL6, CXCL8, IL10 and TNF (TNFα) in plasma samples was assessed using the Luminex technology (Milliplex MAP Human Cytokine/Chemokine/Factor Panel A; Cat no: HCYTA-60 K, Millipore, Darmstadt, Germany). This bead-based assay utilizes fluorescent color-coded beads precoated with capture antibodies, targeting specific cytokines. Thirty-eight plasma samples selected from those used for proteomic studies, after thawing, were centrifuged at 10,000 g for 10 min at 4 °C and 25 μl of undiluted plasmas were run in duplicates per assay. Samples were read with the MAGPIX Systems (Luminex) with xPONENT Software.

### 2.4 Statistical analysis

The Spectronaut Protein Quant Pivot Report was imported into Perseus software v1.6.5.0 (17) for statistical analysis. Proteins with 70% of valid values in at least one group were retained for the analysis prior to any relative quantification. The remaining missing values were imputed column-wise using random values drawn from a normal distribution with a downshift of 1.8 and a width of 0.3, simulating low-abundance values close to the noise level. Prcomp function in R Statistical Software v4.4.1 was used to perform PCA analysis. The first two principal components were plotted. After quantile normalization, the difference in protein expression between each subgroup of elderly individuals and the young group, as well as between males and females in both young and elderly groups, was assessed using Student’s t-test. To reduce the probability of false positive findings deriving from multiple hypothesis testing a permutation-based false discovery rate (FDR) p-value lower than 0.05 was applied, and the artificial within groups variance (s0) was set to 0.1. Data visualizations were performed using R Statistical Software v4.4.1 (18, 19). Differentially expressed proteins were further analyzed by GO (gene ontology)-term and pathway enrichment analysis by ShinyGO webserver (version 0.8) (20). An FDR < 0.05 was used as a significance threshold and fold enrichment scores were taken to assess the enrichment of resulting GO-terms and pathways.

Milliplex Luminex assay data were pre-analyzed by Belysa 1.2.0 software. Sample recovery 80-120% were used for standard curve. ND and LOD values were excluded. Data analysis and graphs were performed by GraphPad Prism software, using unpaired Student’s t-test and 2-way ANOVA.

## 3 Results

**Figure 1:**
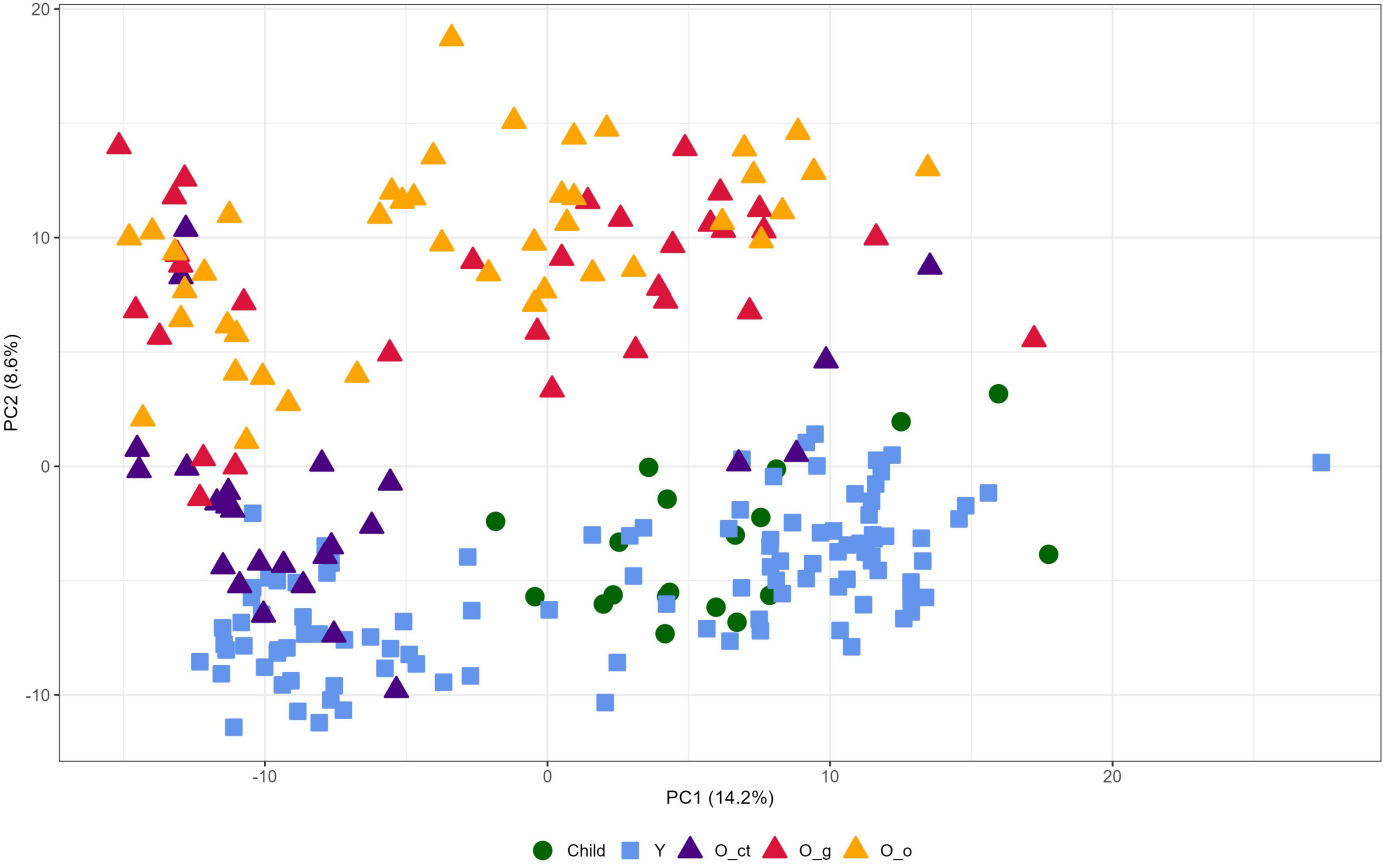
PCA plot of 412 plasma proteins (present in ≥70% of samples in at least one group). PC1 and PC2 explain 14.2% and 8.6% of the total variance, respectively. The Old subgroups are clearly separated from the Young and Child groups except for the O_ct group. Purple Triangle=O_ct group; Red triangle=O_g group; yellow triangle O_o group; blue square=Young group; green circle=Child group.

**Figure 2:**
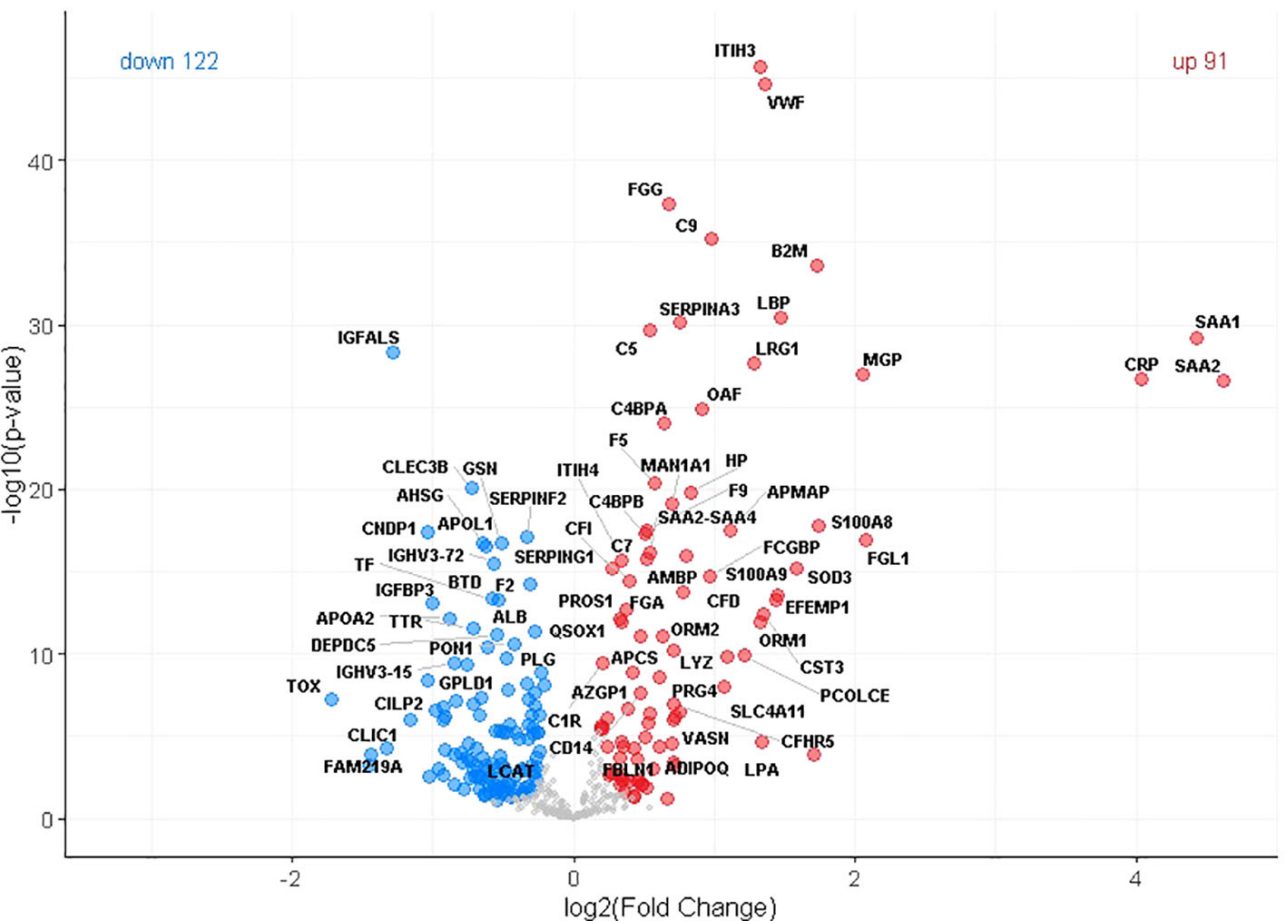
Volcano plot showing differentially expressed plasma proteins between old and young individuals. A total of 412 proteins detected in ≥70% of samples in at least one group were included in the analysis. Compared to the Young group, 91 proteins were significantly upregulated (red circles) and 122 downregulated (blue circles) in the Old group. Differential expression was assessed using a Student’s t-test with statistical thresholds set at s0 = 0.1 and FDR = 0.05.

Our discovery cohort comprised 229 participants distributed across three age groups: children, young adults, and elderly individuals. The primary comparison was conducted between young and elderly participants, while the inclusion of the children group offered a developmental reference point, representing a stage in which regenerative mechanisms are at their peak. The elderly cohort was further stratified into three subcategories: healthy seniors, individuals under geriatric medical care, and patients hospitalized for bone fractures. Using mass-based proteomics, we identified 629 proteins in total, with 412 presented at least 70% of the time in at least one of the three groups: Old, Young, or Child. Principal Component Analysis (PCA) revealed distinct proteome profiles across the young and the old groups (**Figure 1**). Interestingly, in the old group the plasmas from O-ct had a tendency to form a subclaster partially overlapping with plasmas of young and child. To identify proteins that differentiate the Old group from the Young group, we performed a Student’s t-test. Statistically significant differences in protein expression between these two groups were visualized using a volcano plot (**Figure 2**). In total, 213 proteins were found to be dysregulated, including 91 upregulated and 122 downregulated in the Old group compared to the Young group. In this article we focused on proteins upregulated in the Old group. A complete list of these proteins, along with their fold changes relative to the Young and Child group and statistical significance, is provided in **Table 2**.

**Table 2:** Plasma proteins upregulated in the Old group compared to Young and Child.

*Only proteins present in at least 70% plasmas of one of the plasma donor groups and enhanced 1.2 or more folds compared to young are listed.*								
Entrez Gene symbol	UniProt number	Full name	Compared to young			Compared to child		
			*Fold increase*	*Significance*		*Fold increase*	*Significance*	
				*p (-log10)*	*q*		*p (-log10)*	*q*
*SASP (Senescence-Associated Secretory Phenotype)*								
SOD3	P08294	Extracellular superoxide dismutase [Cu-Zn]	3.0	15.2	0.0001	3.0	6.1	0.0009
LPA	P08519	Apolipoprotein(a)	2.5	4.7	0.0001	2.8	2.4	0.0370
CST3	P01034	Cystatin-C	2.5	12.4	0.0001	1.9	2.3	0.0560
VASN	Q6EMK4	Vasorin	1.7	6.2	0.0001	1.0	0.1	0.8800
NID1	P14543	Nidogen-1	1.7	3.2	0.0017	-1.3	0.4	0.5200
LYZ	P61626	Lysozyme C	1.6	10.2	0.0001	1.1	0.4	0.6100
MAN1A1	P33908	Mannosyl-oligosaccharide 1,2-mannosidase IA	1.6	19.1	0.0001	1.4	4.0	0.0560
SHBG	P04278	Sex hormone-binding globulin	1.5	3	0.0035	-1.1	0.2	0.7900
NRP1	O14786	Neuropilin-1	1.4	5	0.0016	1.9	5.5	0.0063
PAK6	Q9NQU5	Serine/threonine-protein kinase PAK 6	1.4	1.3	0.0440	1.8	1.9	0.0890
PCSK9	Q8NBP7	Proprotein convertase subtilisin/kexin type 9	1.3	2.3	0.0180	1.7	2.5	0.0580
AZGP1	P25311	Zinc-alpha-2-glycoprotein	1.3	8.8	0.0008	1.7	9.3	0.0016
POSTN	B1ALD9	Periostin	1.3	1.4	0.0400	-2.5	2.9	0.0250
SERPING1	P05155	Plasma protease C1 inhibitor	1.3	14.5	0.0004	1.1	1.1	0.4600
ALCAM	Q13740	CD166 antigen	1.3	1.6	0.0420	1.1	0.2	0.7900
IGFBP6	P24592	Insulin-like growth factor-binding protein 6	1.3	3.7	0.0326	2.9	14.0	0.0206
HGFAC	Q04756	Hepatocyte growth factor activator	1.2	4.4	0.0260	1.2	2.4	0.1800
*SASP/Hemostasis. Blood coagulation control*								
FGL1	Q08830	Fibrinogen-like protein 1	4.2	16.9	0.0001	4.4	5.4	0.0010
VWF	P04275	von Willebrand factor	2.6	44.7	0.0001	6.2	24.5	0.0001
FGG	P02679	Fibrinogen gamma chain	1.6	37.4	0.0001	1.8	15.7	0.0001
F5	P12259	Coagulation factor V	1.5	20.4	0.0001	1.4	5.5	0.0330
F9	P00740	Coagulation factor IX	1.4	17.5	0.0001	2.1	17.9	0.0001
PROS1	P07225	Vitamin K-dependent protein S	1.3	12.2	0.0020	1.2	1.5	0.4100
FGA	P02671	Fibrinogen alpha chain	1.3	12.7	0.0008	1.6	10.9	0.0018
FGB	P02675	Fibrinogen beta chain	1.2	6.1	0.0210	1.3	5.3	0.0600
*DAMPS (Damage-Associated Molecular Patterns)*								
KRT5	P13647	Keratin, type II cytoskeletal 5	3.3	3.9	0.0001	1.9	0.7	0.3000
S100A8	P05109	Protein S100-A8	3.3	17.8	0.0001	-1.3	0.7	0.4000
S100A9	P06702	Protein S100-A9	2.7	13.5	0.0001	-1.4	0.8	0.3200
EFEMP1	Q12805	EGF-containing fibulin-like extracellular matrix protein 1	2.7	13.3	0.0001	4.4	10.3	0.0001
PCOLCE	Q15113	Procollagen C-endopeptidase enhancer 1	2.3	9.9	0.0001	2.4	3.4	0.0160
CCDC112	Q8NEF3	Coiled-coil domain containing 112	1.6	3.4	0.0015	2.1	3.1	0.0260
KRT1	P04264	Keratin, type II cytoskeletal 1	1.6	1.2	0.0360	1.1	0.1	0.8700
ZNF280C	Q8ND82	Zinc finger protein 280C	1.6	6	0.0002	2.5	6.9	0.0009
PRG4	Q92954	Proteoglycan 4	1.5	8.6	0.0001	1.5	2.4	0.0930
HSP90B1	P14625	Endoplasmin	1.3	4.3	0.0036	1.3	1.7	0.1900
*Complement activation*								
C9	P02748	Complement component C9	2.0	35.2	0.0001	1.8	8.7	0.0018
CFD	P00746	Complement factor D	1.7	13.7	0.0001	3.9	17.8	0.0001
CFHR5	Q9BXR6	Complement factor H-related protein 5	1.6	7	0.0001	2.2	6.6	0.0017
C4BPA	P04003	C4b-binding protein alpha chain	1.6	24	0.0001	1.2	2.1	0.2200
C5	P01031	Complement C5	1.5	29.6	0.0001	1.6	12.6	0.0016
C4BPB	P20851	C4b-binding protein beta chain	1.4	17.3	0.0001	1.4	5.3	0.0490
C7	P10643	Complement component C7	1.4	15.8	0.0001	1.1	1.1	0.4200
MASP2	O00187	Mannan-binding lectin serine protease 2	1.4	2.2	0.0150	1.0	0	0.9700
C4B_2	P0C0L5	Complement C4-B	1.3	4.3	0.0080	1.3	2.3	0.1300
C2	P06681	Complement C2	1.3	4.6	0.0082	1.2	2.1	0.2000
C6	P13671	Complement component C6	1.2	5.5	0.0370	1.1	1.3	0.4400
CFI	P05156	Complement factor I	1.2	15.2	0.0036	1.1	2.0	0.3800
C1QC	P02747	Complement C1q subcomponent subunit C	1.2	2.8	0.0310	1.2	0.9	0.4100
C4A	P0C0L4	Complement C4-A	1.2	2.7	0.0420	1.3	1.6	0.2200
C1R	P00736	Complement C1r subcomponent	1.2	9.5	0.0230	1.3	6.5	0.0730
*Inflammatory response*								
SAA2	P0DJI9	Serum amyloid A-2 protein	24.6	26.6	0.0001	15.7	6.6	0.0001
SAA1	P0DJI8	Serum amyloid A-1 protein	21.5	29.2	0.0001	29.5	9.8	0.0001
CRP	P02741	C-reactive protein	16.4	26.7	0.0001	24.0	10.6	0.0001
B2M	P61769	Beta-2-microglobulin	3.3	33.6	0.0001	4.3	14.6	0.0001
LBP	P18428	Lipopolysaccharide-binding protein	2.8	30.5	0.0001	3.1	11.5	0.0001
ORM1	P02763	Alpha-1-acid glycoprotein 1	2.5	12	0.0001	2.2	3.5	0.0170
ITIH3	Q06033	Inter-alpha-trypsin inhibitor heavy chain H3	2.5	45.7	0.0001	2.2	11.4	0.0001
HP	P00738	Haptoglobin	1.8	19.8	0.0001	2.3	8.7	0.0001
AMBP	P02760	Protein AMBP	1.7	16	0.0001	2.3	11.9	0.0001
RARRES2	Q99969	Retinoic acid receptor responder protein 2	1.7	6.5	0.0001	2.2	4.7	0.0062
SERPINA3	P01011	Alpha-1-antichymotrypsin	1.7	30.1	0.0001	1.7	9.2	0.0016
ADIPOQ	Q15848	Adiponectin	1.6	4.5	0.0004	1.0	0.1	0.9200
ORM2	P19652	Alpha-1-acid glycoprotein 2	1.5	11.1	0.0001	1.2	1.1	0.3700
CD14	P08571	Monocyte differentiation antigen CD14	1.4	7.6	0.0005	1.4	3.2	0.0790
APCS	P02743	Serum amyloid P-component	1.4	11.1	0.0002	2.0	15.4	0.0000
ITIH4	Q14624	Inter-alpha-trypsin inhibitor heavy chain H4	1.3	15.7	0.0008	1.2	3.6	0.1700
APOC4-APOC2	P02655	Apolipoprotein C-II	1.3	2.3	0.0240	2.2	6.6	0.0018
APOH	P02749	Beta-2-glycoprotein 1	1.2	2.8	0.0350	1.4	1.7	0.1600
*Enhanced proteins often dealing with tissue homeostasis but without the identification of a well-defined pathway*								
MGP	P08493	Matrix Gla protein	4.1	27	0.0001	6.2	21.6	0.0001
LRG1	P02750	Leucine-rich alpha-2-glycoprotein	2.4	27.6	0.0001	2.5	10.2	0.0001
FCGBP	Q9Y6R7	IgGFc-binding protein	2.0	14.7	0.0001	2.2	5.7	0.0032
OAF	Q86UD1	Out at first protein homolog	1.9	24.9	0.0001	2.3	14.6	0.0001
DCSTAMP	Q9H295	Dendritic cell-specific transmembrane protein	1.4	2.1	0.0150	1.6	1.5	0.1500
QSOX1	O00391	Sulfhydryl oxidase 1	1.3	11.9	0.0016	1.2	2.8	0.1900
LCP1	P13796	Plastin-2	1.3	2.6	0.0200	-1.2	0.7	0.4500
PIGR	P01833	Polymeric immunoglobulin receptor	1.3	2.3	0.0220	1.6	1.6	0.1300
LYVE1	Q9Y5Y7	Lymphatic vessel endothelial hyaluronic acid receptor 1	1.3	2.0	0.0330	1.9	3.7	0.0210

**Figure 3:**
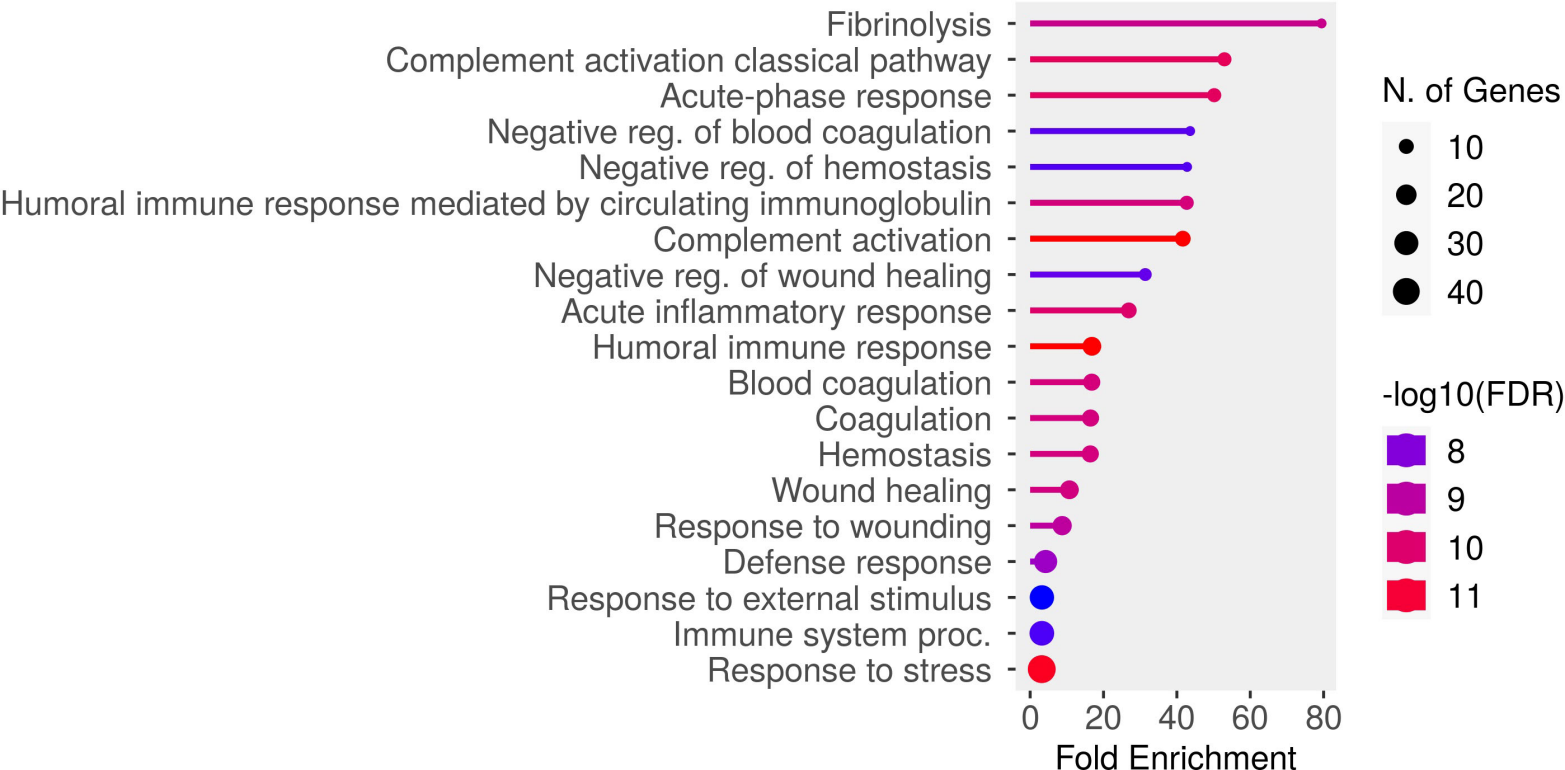
Gene Ontology Biological Process enrichment analysis of proteins upregulated in the Old group relative to the Young group. The analysis was performed using ShinyGO tool, with a significance threshold of FDR ≤ 0.05 and the entire human database as a background. The most significantly enriched GO terms are displayed.

In **Table 2**, proteins are grouped based on their known involvement in specific pathways or cellular functions. In Old group-derived plasma, we observed an increased concentration of proteins and molecules classified as SASPs in the SASP atlas (see also **Supplementary File 1**) (21), including several linked to the dysregulation of homeostasis, blood coagulation, and Damage-Associated Molecular Patterns (DAMPs). This increase in SASPs and DAMPs may contribute to the significant activation of the complement system and the presence of inflammatory proteins. To further explore the pathways associated with differentially expressed proteins, we performed Gene Ontology (stem GO) enrichment analysis of biological processes (BP). Among the most significantly enriched terms for upregulated proteins in the Old group were complement activation, complement activation (classical pathway), acute-phase response and acute inflammatory response (**Figure 3**).

A central role among the identified pathways and functions is fulfilled by the inflammatory response. Several cytokines and growth factors are known to trigger and control inflammation. However, it was not feasible to reveal possible differences existing in the plasma concentration of this type of molecules because, due to their low concentration and the mass technology limitations, they were not among the 612 identified proteins.

We already reported in a recent article of ours an enhancement in the old plasmas compared to young of some representative cytokines and growth factors: Growth/Differentiation Factor (GDF-15), Granulocyte-Macrophage Colony-Stimulating Factor (CSF2), C-C motif chemokine 3 (CCL3) and cytokines stimulating proliferation and maturation of different types of leukocytes as IL3. Interestingly the enhanced bioactive molecules included also molecules (IL1RN, IL18BP, IL27) with an anti-inflammatory property or an inhibitory activity on pro-inflammatory cytokines suggesting a finer tuning of inflammation (6).

To investigate possible variations in the concentration of some additional cytokines we adopted a Milliplex Luminex assay. Results are shown in **Table 3**. The enhanced cytokines and growth factors included classical pro-inflammatory cytokines and chemokines such as IL6, CXCL8 (IL8), and TNF (TNFα). Only no statistically significant slight enhancements were observed for IL1A, the anti-inflammatory IL10, and the interferon-gamma (IFNG) which increases during a viral infection and in some circumstances, it can act as a pro-inflammatory molecule. In contrast, an increase of IL1B, a proinflammatory cytokine also involved in modulation of autoimmune inflammation, was observed in the young plasmas.

**Table 3:** Cytokine expression in the Old group compared to Young.

Entrez Gene symbol	UniProt number	Full name	Fold increase in old	Significance *p* value	# of tested young plasmas	# of tested old plasmas
IL1A	P01583	Interleukin-1 alpha	1.1	0.891	4	5
IL1B	P01584	Interleukin-1 beta	-1.6	0.299	7	13
IL6	P05231	Interleukin-6	7.9	0.023	10	28
CXCL8 (IL8)	P10145	Interleukin-8	1.9	0.022	10	28
IL10	P22301	Interleukin-10	-1.1	0.777	10	27
TNF	P01375	Tumor necrosis factor-alpha	1.7	0.022	10	28
IFNG	P01579	Interferon gamma	1.1	0.634	10	28

The segments of different immunoglobulin chains that were enhanced or reduced in old plasmas compared to young are shown in **Table 4**. Changes in immunoglobulin chain concentrations may reflect an alteration of specific antibody production, and possibly a distorted formation of immune complexes, against endogenous and/or exogenous antigens. It is to note that in most cases in the old plasmas there is a chain concentration reduction and that 2/3 of the observed reductions concern kappa and lambda chain segments. With aging the antibody production by B cells become less efficient (immunesenescence) (22, 23). This pattern is consistent with a pro-inflammatory environment characteristic of aging (inflammaging), involving both immune systems: the innate (e.g., complement activation) and the adaptive one (e.g., immunoglobulins).

**Table 4:** Immunoglobulin chain expression in the Old group compared to Young.

Entrez Gene symbol	Antibody chain	UniProt full name	*Fold increase*	*Significance*	
				*p (-log10)*	*q*
P23083	IGHV1-2	Immunoglobulin heavy variable 1-2	-1.2	2.7	0.0294
A0A0C4DH33	IGHV1-24	Immunoglobulin heavy variable 1-24	-1.4	2.0	0.0176
P01780	IGHV3-7	Immunoglobulin heavy variable 3-7	-1.2	6.9	0.0102
A0A0B4J1V0	IGHV3-15	Immunoglobulin heavy variable 3-15	-1.8	9.5	0.0001
A0A4W8ZXM2	IGHV3-72	Immunoglobulin heavy variable 3-72	-1.5	15.5	0.0001
A0A0B4J1U7	IGHV6-1	Immunoglobulin heavy variable 6-1	-1.5	5.3	0.0008
P01860	IGHG3	Immunoglobulin heavy constant gamma 3	-1.3	2.9	0.0175
P01861	IGHG4	Immunoglobulin heavy constant gamma 4	-1.3	1.4	0.0443
P01880	IGHD	Immunoglobulin heavy constant delta	-2.7	3.9	0.0001
P01871	IGHM	Immunoglobulin heavy constant mu	-1.6	7.3	0.0001
A0A0C4DH67	IGKV1-8	Immunoglobulin kappa variable 1-8	-1.2	3.1	0.0298
P04430	IGKV1-16	Immunoglobulin kappa variable 1-16	-1.3	1.9	0.0342
P01601	IGKV1D-16	Immunoglobulin kappa variable 1D-16	-1.7	2.5	0.0036
P06310	IGKV2-30	Immunoglobulin kappa variable 2-30	-1.5	10.4	0.0001
A0A075B6R9	IGKV2D-24	Probable non-functional immunoglobulin kappa variable 2D-24	-1.6	6.2	0.0002
P01624	IGKV3-15	Immunoglobulin kappa variable 3-15	-1.7	9.4	0.0001
P01619	IGKV3-20	Immunoglobulin kappa variable 3-20	-1.2	5.7	0.0071
P04433	IGKV3D-11	Immunoglobulin kappa variable 3D-11	-1.2	5.2	0.0181
A0A0C4DH25	IGKV3D-20	Immunoglobulin kappa variable 3D-20	-1.2	3.7	0.0231
P06312	IGKV4-1	Immunoglobulin kappa variable 4-1	-1.2	2.9	0.0300
A0A0C4DH24	IGKV6-21	Immunoglobulin kappa variable 6-21	-1.4	3.4	0.0035
A0A0A0MT36	IGKV6D-21	Immunoglobulin kappa variable 6D-21	-1.3	1.6	0.0438
P01834	IGKC	Immunoglobulin kappa constant	-1.2	5.6	0.0126
A0A0B4J1U3	IGLV1-36	Immunoglobulin lambda variable 1-36	-1.6	3.2	0.0021
A0A087WSX0	IGLV5-45	Immunoglobulin lambda variable 5-45	-1.9	3.1	0.0009
P04211	IGLV7-43	Immunoglobulin lambda variable 7-43	-1.3	4.8	0.0037
A0A075B6I0	IGLV8-61	Immunoglobulin lambda variable 8-61	-1.3	2.7	0.0131
P01715	IGLV3-1	Immunoglobulin lambda variable 3-1	1.4	1.9	0.0164
A0A075B6I9	IGLV7-46	Immunoglobulin lambda variable 7-46	-1.5	3.3	0.0028

**Figure 4:**
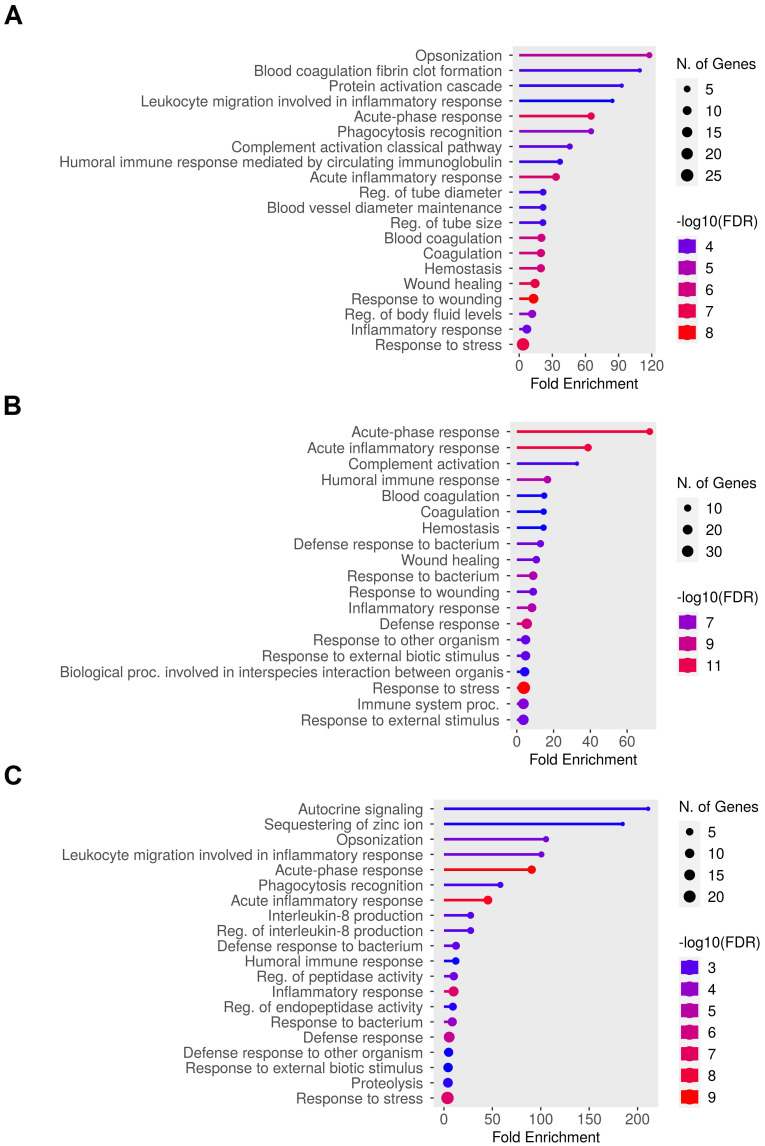
Differences in plasma protein expression among the Old subgroups. The Student’s t-test was performed to highlight differences between each Old subgroup and the Young group (Student’s t-test: Old subgroups vs. Young, s0 = 0.1, FDR = 0.05). Panel **(A)** Gene Ontology Biological Process enrichment analysis of the 47 significantly upregulated proteins in the O-ct group; Panel **(B)** Gene Ontology Biological Process enrichment analysis of the 61 significantly upregulated proteins in the O-g group; Panel **(C)** Gene Ontology Biological Process enrichment analysis of the 41 significantly upregulated proteins in the O-o group. The analysis was performed using ShinyGO tool, with a significance threshold of FDR ≤ 0.05 and the entire human database as a background. The most significantly enriched GO terms are displayed.

We then focused on differences existing among the subgroups in the Old group. The Student’s t-test was used to highlight differences between each Old subgroup and the Young group. The analysis identified 47 significantly upregulated proteins in the O-ct group, 61 in the O-g group, and 41 in the O-o group compared to the Young group. GO enrichment analysis of these proteins (**Figures 4A–C**) revealed common significant pathways, including acute-phase response, acute inflammatory response, inflammatory response, humoral immune response, and response to stress. These findings suggest that, regardless of the specific subgroup, older individuals exhibit a persistent activation of inflammatory responses, coagulation processes, and humoral immunity compared to younger individuals.

**Figure 5:**
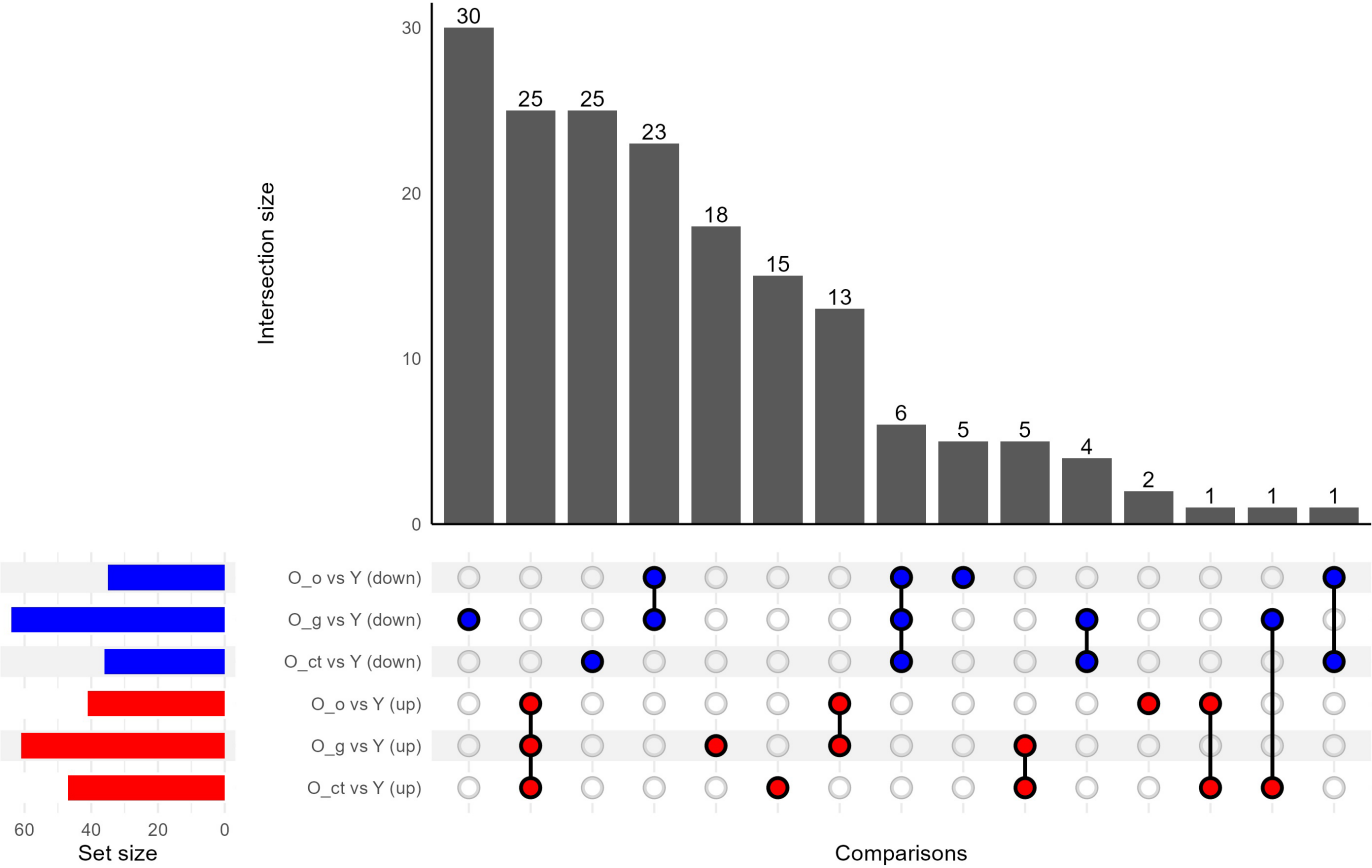
UpSet plot of Differentially Regulated Plasma Proteins. Horizontal bars on the left represent the number of proteins that are differentially regulated in each of the three comparisons O_o vs. Young, O_g vs. Young, and O_ct vs. Young - with the proteins first grouped by those that are downregulated and then by those that are upregulated. Vertical bars indicate the size of each intersection, with the connected dots below each bar specifying the combinations of comparisons in which the respective set of proteins is differentially regulated. Single dots represent proteins uniquely altered in a single comparison, while connected dots denote proteins shared among two or more comparisons. This figure highlights the overlapping and distinct signatures of plasma protein regulation between elderly subgroups and young donors.

**Figure 6:**
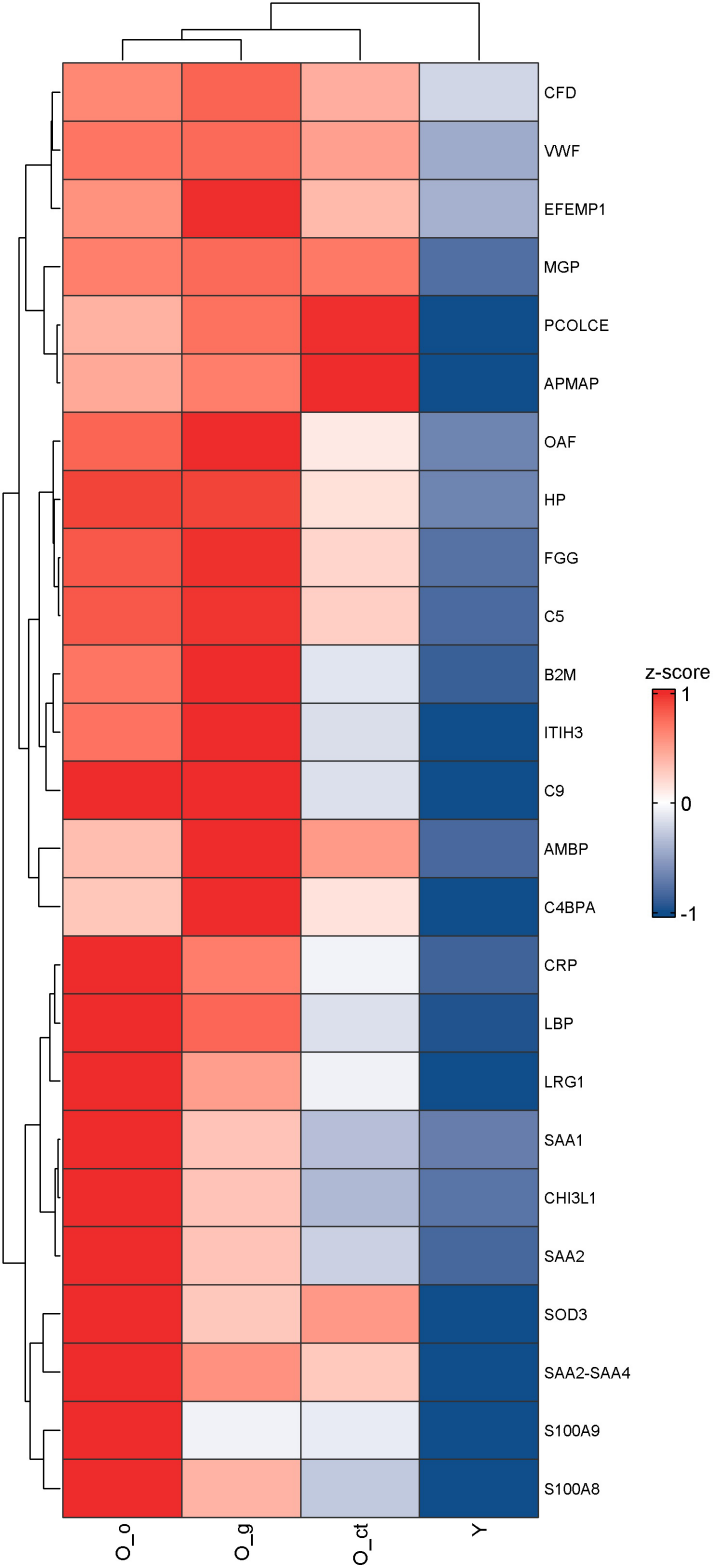
The heatmap displays the expression profiles of the 25 plasma proteins consistently elevated across all three Old subgroups. Red indicates upregulation, while blue indicates downregulation. Variations associated with life-experience and recent traumatic events are evident. A distinct cluster of proteins (bottom 10 rows) shows marked upregulation in the orthopedic (O_o) subgroup.

By data analysis, 25 proteins were found to be upregulated - albeit to varying degrees - in all three elderly subgroups compared to the young adult group, as presented by UpSet plot (**Figure 5**). All those 25 proteins are associated with inflammatory processes. Functionally, they are related to immune response, complement activation, coagulation cascades, and protein-lipid complexes. We therefore propose these 25 consistently upregulated proteins as a plasma proteomic signature of aging whose expression pattern was illustrated as heatmap in **Figure 6**. Despite the shared upregulation, the extent of expression varies among subgroups, revealing distinct protein clusters that appear to be modulated by life-experience factors. The superposition of a dramatic event - such as a bone fracture, typical of the orthopedic subgroup - on this common inflammatory background, elicits a strong acute phase response, reflecting the body’s reaction to recent trauma. A specific cluster of ten proteins (depicted in the bottom ten rows of **Figure 6**) was highly upregulated in this subgroup, suggesting an additional layer of inflammation linked to the injury, which occurred just a few days prior to plasma sampling. Among these, SAA1, SAA2, CRP and CHI3L1 exhibit particularly elevated plasma concentrations. For SAA1, SAA2, and CRP, the fold increase was striking - ranging from nearly 60-fold to over 90-fold compared to young controls. A specific comment should be made for the CHI3L1 (Chitinase-3-like protein 1). This protein was not included in **Table 2** since it is virtually undetectable in the Child group and does not reach the 70% presence threshold in the plasma samples of the Young and Old groups. However, when the analysis was performed considering separately the old plasmas subgroup a completely different pathway of expression was observed. The CHI3L1was present in 93% of the O-o group with a 10.9-fold increase compared to young plasmas, in 65% of the O-g samples with a 3.7-fold increase compared to young, and in 23% of the O-ct samples with a 2.0-fold increase compared to young. This suggests that the CHI3L1 protein is one of the major components of the acute phase response to a dramatic event such as a fracture, but its role is less relevant in a chronic inflammatory condition as the one observed in elderly people. These results are in line with findings from Lehallier et al. (9, 24), who showed that individuals undergoing regular aerobic exercise exhibit a proteomic profile reflective of a younger biological age compared to sedentary individuals, underscoring the influence of life-experience on aging-associated molecular signatures.

**Figure 7:**
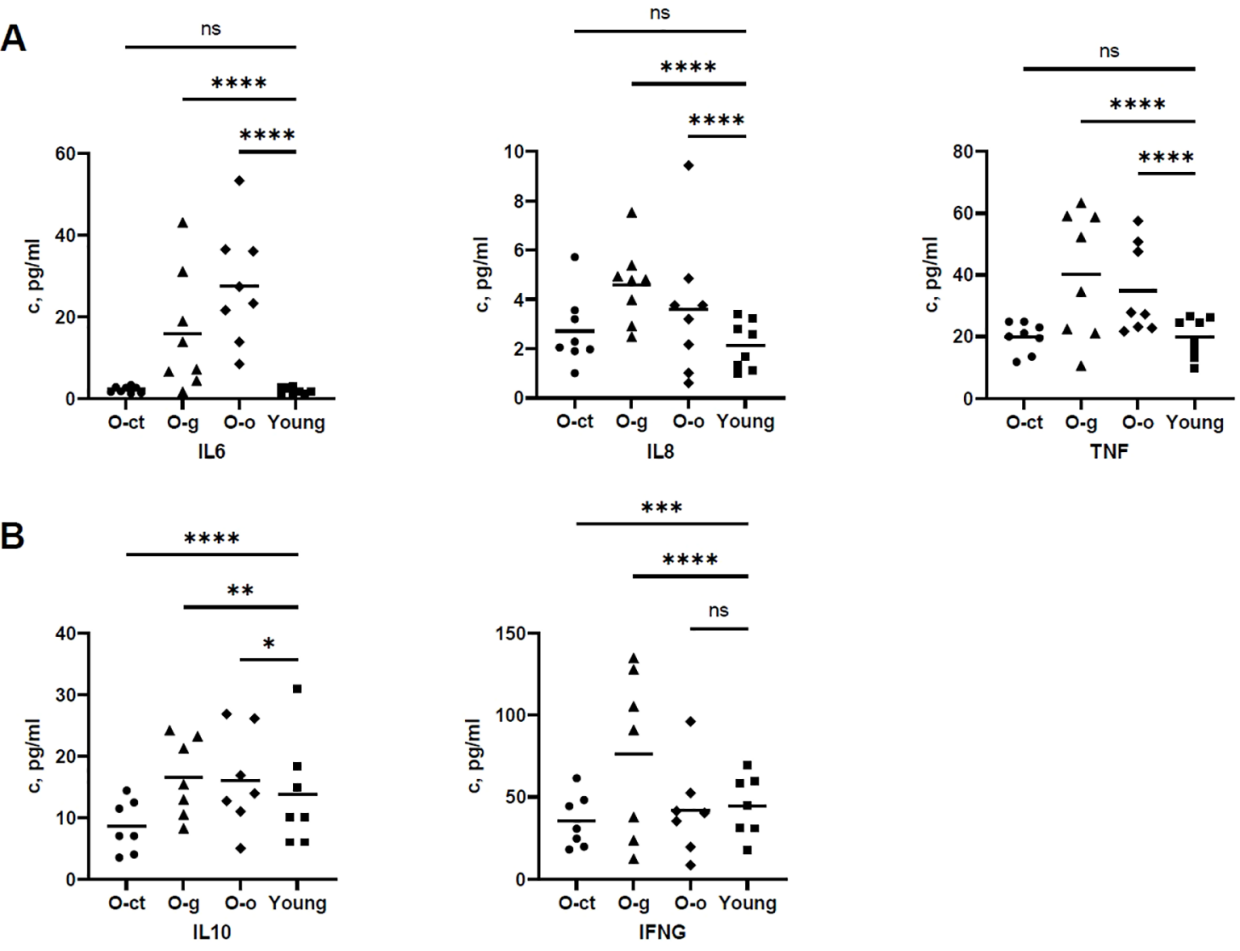
Comparison of plasma cytokine and chemokine concentrations in the Old subgroups and in the Young group. The graph compares concentrations present in plasmas of O-ct, O-g, and O-o to Young group; Panel **(A)** Concentrations of classical pro-inflammatory cytokines IL6, chemokine IL8 and TNF (TNFα). Significant increase is reported in plasmas of O-g and O-o compared to Young group whereas no differences existed between the O-ct and the Young group; Panel **(B)** Concentrations of the anti-inflammatory IL10 and of the antiviral IFNG (IFNγ). A highly significant reduction of IL10 concentration was observed in the O-ct subgroup compared to the Young group. The concentration of IFNG was highest in the O-g subgroup. (*p ≤ 0.05, **p ≤ 0.01,***p ≤ 0.001 and ****p ≤ 0.0001).

Differences were also observed when we determined plasma cytokine concentrations in the different subgroups. In **Figure 7A** it is shown the comparison of the classical pro-inflammatory factors IL6, CXCL8 (IL8) and TNF (TNFα) concentrations. Interestingly, while a significant increase was observed in plasmas of O-g and O-o compared to young, no differences were observed between the O-ct and the young groups thus confirming the importance of an active life-experience to counteract the onset of an inflammatory condition during aging. Interestingly, the O-ct subgroup presented the lowest level of the anti-inflammatory IL10 compared to both the other two subgroups and the Young group, whereas the highest expression of the antiviral IFNG (IFNγ) was observed in the O-g subgroup, a group of patients possibly at higher risk of nosocomial infections because of the long hospitalization (**Figure 7B**). The concentration of GM-CSF was found to be below the assay’s threshold and therefore could not be reliably quantified.

The old subgroups differed also regarding the level of immune senescence. When the comparison with the immunoglobulins significantly expressed in the young plasmas was made for each subgroup, we observed in the O-ct a percentage of chains under the significance threshold of 27% with an average reduction of 1.5 folds for the 73% chains above the threshold. On the contrary, the percentages of chains below the significance threshold for the O-o and O-g subgroups were 60% and 74% respectively with a fold reduction of 1.8 and 2.2 folds for the 40% and 26% chains remaining above the threshold. Thus, confirming once more the beneficial effect of a healthy life.

**Figure 8:**
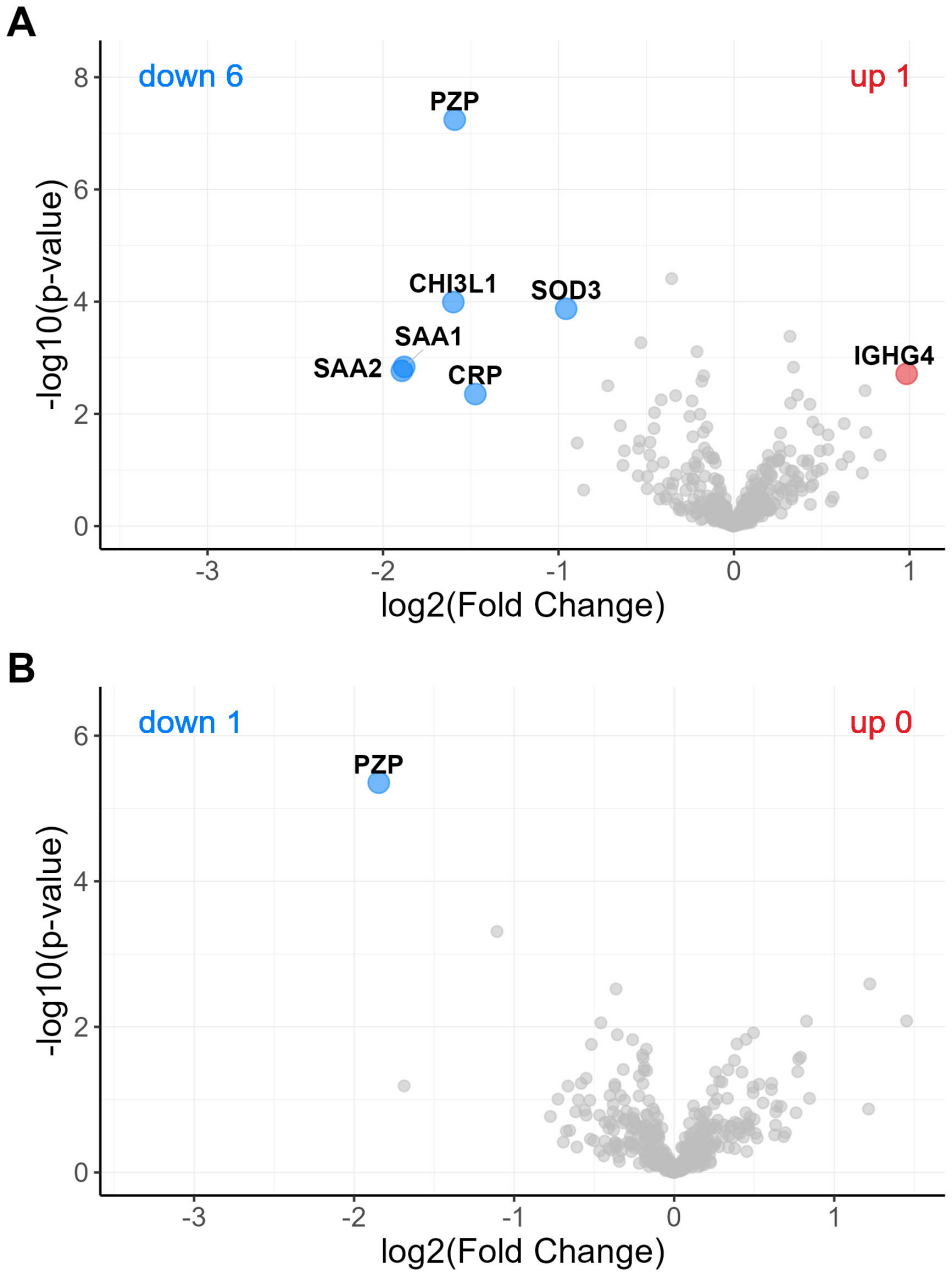
Volcano plot showing differentially expressed plasma proteins between old males and old females. The analysis included 395 proteins detected in ≥70% of samples in at least one of the two groups. Panel **(A)** Proteins significantly upregulated in three subgroups of males are shown as red circles, and those upregulated in females as blue circles. Panel **(B)** Proteins significantly downregulated in two subgroups of males (O-ct and O-g) are shown as blue circles. Statistical analysis was performed using a Student’s t-test with thresholds of s0 = 0.1 and FDR = 0.05.

To investigate potential sex-related differences in the expression of plasma proteins, we first compared male and female samples within the entire old group using Student’s t-test, and visualized the results as a volcano plot (**Figure 8A**). In this initial comparison—which included all three old subgroups - we observed higher concentrations of Pregnancy Zone Protein (PZP) and Superoxide Dismutase 3 (SOD3) in female plasmas. Additionally, elevated levels of SAA1, SAA2, CRP, and CHI3L1 were also detected. However, we hypothesized that the increased levels of these four acute-phase proteins might not reflect intrinsic sex differences but rather result from the uneven distribution of females and males in the orthopedic subgroup, which is characterized by a marked inflammatory response due to recent fractures. To address this, we repeated the analysis including only the O-ct and O-g subgroups - excluding the orthopedic cases (**Figure 8B**). In this refined comparison, PZP emerged as the only protein significantly more abundant in elderly female plasmas, while no other proteins showed significant differences between males and females. Importantly, no proteins were found to be selectively elevated in male plasmas.

**Figure 9:**
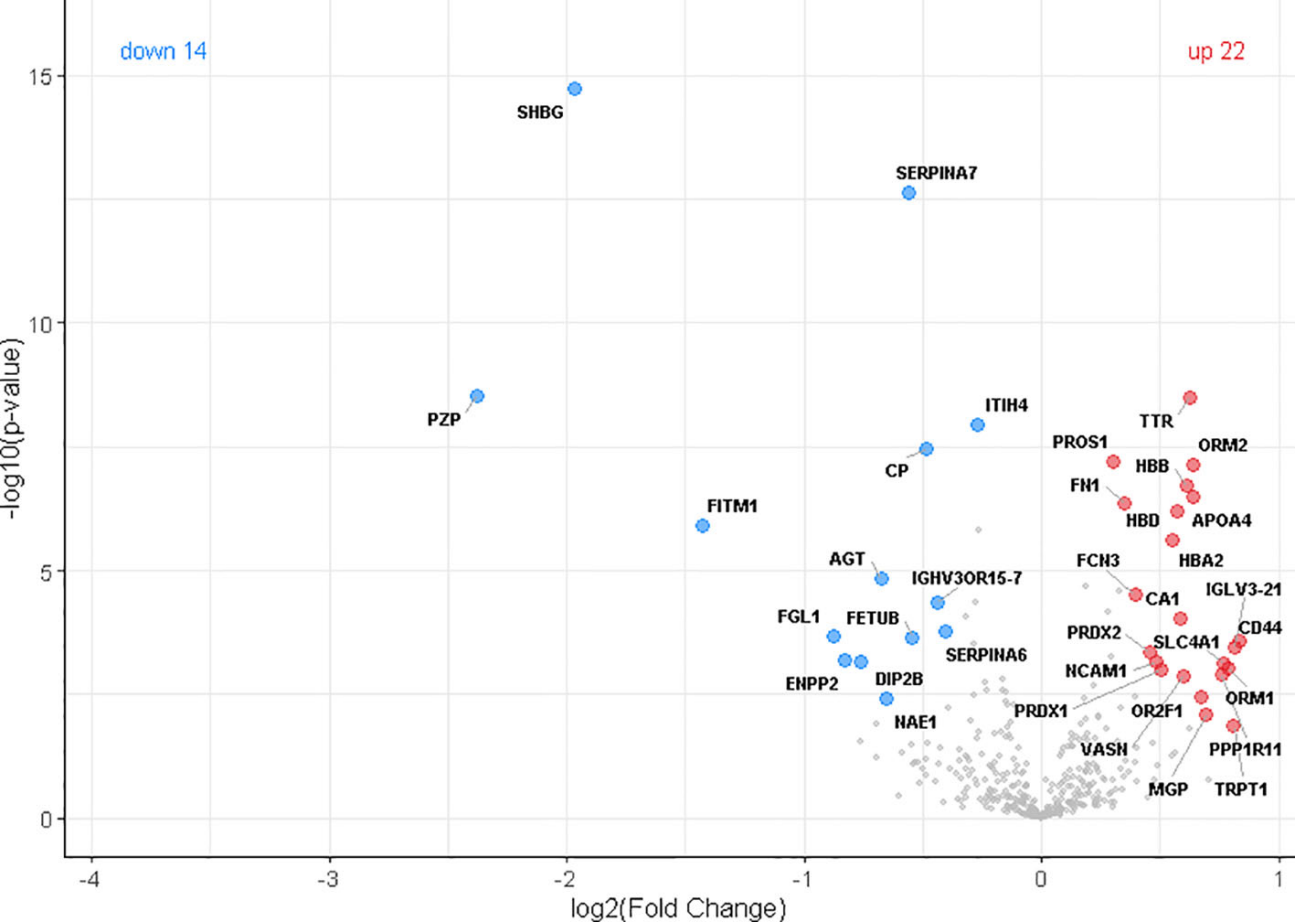
Volcano plot showing differentially expressed plasma proteins between young males and young females. Proteins detected in ≥70% of samples in at least one of the two groups were included in the analysis. Red circles indicate proteins upregulated in males, and blue circles indicate proteins upregulated in females. Significantly modulated proteins were identified using a Student’s t-test with s0 = 0.1 and FDR = 0.05. All significantly modulated proteins are listed in **Table 5**.

In contrast to the Old group, several significant sex-based differences in both the nature and concentration of plasma proteins were observed between young males and females (**Figure 9**). A detailed list of differentially expressed proteins is provided in **Table 5**.

**Table 5:** Proteins enhanced in young female and young male plasmas.

*Only proteins enhanced 1.2 or more folds are listed.*					
Entrez Gene symbol	UniProt number	Full name	*Fold increase*	*Significance*	
				*p(-log10)*	*q*
*Proteins enhanced in young females*					
P20742	PZP	Pregnancy zone protein	5.2	8.5	<0.001
I3L145	SHBG	Sex hormone-binding globulin	3.9	14.7	<0.001
H0YL77	FITM1	Fat storage-inducing transmembrane protein 1	2.7	5.9	<0.001
Q08830	FGL1	Fibrinogen-like protein 1	1.8	3.7	0.0058
E7EUF1	ENPP2	Ectonucleotide pyrophosphatase/phosphodiesterase 2	1.8	3.2	0.0083
Q9P265	DIP2B	Disco-interacting protein 2 homolog B	1.7	3.1	0.0111
P01019	AGT	Angiotensinogen	1.6	4.8	0.0056
Q13564	NAE1	NEDD8-activating enzyme E1 regulatory subunit	1.6	2.4	0.0351
P05543	SERPINA7	Thyroxine-binding globulin	1.5	12.6	<0.001
Q9UGM5	FETUB	Fetuin-B	1.5	3.7	0.0182
P00450	CP	Ceruloplasmin	1.4	7.4	0.0053
A0A075B7D8	IGHV3OR15-7	Immunoglobulin heavy variable 3/OR15-7 (pseudogene)	1.4	4.3	0.0271
P08185	SERPINA6	Corticosteroid-binding globulin	1.3	3.8	0.0355
Q14624	ITIH4	Inter-alpha-trypsin inhibitor heavy chain H4	1.2	7.9	0.0488
*Proteins enhanced in young males*					
H0Y2P0	CD44	CD44 antigen	1.8	3.6	0.0054
P80748	IGLV3-21	Immunoglobulin lambda variable 3-21	1.8	3.4	0.0053
F5H6B6	TRPT1	2’-phosphotransferase	1.8	1.8	0.0473
P02763	ORM1	Alpha-1-acid glycoprotein 1	1.7	3.0	0.0102
P02730	SLC4A1	Band 3 anion transport protein	1.7	3.1	0.0105
O60927	PPP1R11	E3 ubiquitin-protein ligase PPP1R11	1.7	2.9	0.0128
P08493	MGP	Matrix Gla protein	1.6	2.1	0.0427
Q13607	OR2F1	Olfactory receptor 2F1	1.6	2.5	0.0326
P06727	APOA4	Apolipoprotein A-IV	1.6	6.5	0.0034
P19652	ORM2	Alpha-1-acid glycoprotein 2	1.6	7.1	0.0020
P02766	TTR	Transthyretin	1.5	8.5	0.0008
P68871	HBB	Hemoglobin subunit beta	1.5	6.7	0.0035
Q6EMK4	VASN	Vasorin	1.5	2.9	0.0292
P00915	CA1	Carbonic anhydrase 1	1.5	4.0	0.0113
P02042	HBD	Hemoglobin subunit delta	1.5	6.2	0.0058
P69905	HBA2	Hemoglobin subunit alpha	1.5	5.6	0.0055
Q06830	PRDX1	Peroxiredoxin-1	1.4	3.0	0.0332
P13591	NCAM1	Neural cell adhesion molecule 1	1.4	3.1	0.0348
P32119	PRDX2	Peroxiredoxin-2	1.4	3.4	0.0343
O75636	FCN3	Ficolin-3	1.3	4.5	0.0338
P02751-1	FN1	Fibronectin,	1.3	6.4	0.0282
A0A0S2Z4L3	PROS1	Vitamin K-dependent protein S	1.2	7.2	0.0343

The Pregnancy Zone Protein (PZP) is the only protein enhanced in both old and young female plasmas. Compared to old and young male plasmas, in female plasmas of the same age group PZP is enhanced more than 3 and 5 times respectively. In young females, the second protein with a higher concentration, after PZP, compared to males (3.9-fold increase) is the Sex Hormone-Binding Globulin (SHBG).

## 4 Discussion

Plasma proteins are suitable targets to investigate “omics” changes occurring with age since they are final effectors of many physio- pathological processes and pathways. Technological progresses, such as SOMAscan assay and mass spectrometry (MS)-based proteomics, made possible the valuation of thousands of proteins in plasma as biomarkers to investigate health compromise and functional decline associated with aging.

Several studies identified proteins in human tissues and biological fluids that change with age. Among the main published articles, Menni et al., to elucidate the proteomic features of aging-related phenotypes, tested plasma by the SOMAscan assay. Eleven proteins were associated with chronological age (25). Santos-Lozano et al. performed a comparative analysis of plasma proteins of healthy female centenarians (age range 100–103 years) and control individuals who died from a major age -related disease before the expected life expectancy (age range: 67–81 years). They found that the expression of 49 proteins and 86 pathways differed between the two groups. Overall, healthy centenarians presented a distinct expression of proteins/pathways that reflect a lower pro-inflammatory status, less autoimmunity, and a preserved humoral immune response (7). Lehallier et al. measured 2,925 proteins from 4,263 plasmas of young adults to nonagenarians (18–95 years old) and developed a new bioinformatics approach to uncover age changes in the human plasma proteome. They observed distinct waves of changes in the fourth, seventh and eighth decades of life. The new approach to the study of aging led to the identification of potential targets for the treatment of age-related diseases (9, 24). In other cases genetic variations in plasma proteins were investigated in thousands of mostly healthy individuals as predictive factors of specific disease (26–29).

Moaddel et al. performed a detailed literature search for proteomic analysis in different tissues, including plasma humans and other species (30). Inclusion criteria for the articles were healthy individuals and proteomics results published on or after 2010. Twelve manuscripts covered human plasma data (7, 9, 13, 21, 25, 31–37). Of the initially identified proteins, 232 were age-associated, and the expression in plasma and other matrices increased with age. In a similar study Johnson et al. examined 32 articles and identified 1,128 proteins that differed by age groups in two or more studies, and only 32 proteins that differed in at least five independent studies (38). The study of an array of proteins, rather than a single biomarker, should allow a better comprehension of mechanisms underlying aging.

In a proteomic analysis of almost a thousand plasmas Tanaka et al. identified 506 proteins over-represented with age of which 33.5% and 45.3%, were associated with mortality and multimorbidity, respectively (39). Some of the enriched proteins were associated with inflammation and extracellular matrix as well as senescence-associated secretory proteins. Proteins enhanced in plasma with aging included proteins secreted by senescent cells also in other studies. Cellular senescence is a stress response that induces a permanent cell cycle arrest and the secretion of a complex association of proteins defined as the Senescence-Associated Secretory Phenotype (SASP), eventually leading to the release of inflammatory cytokines with autocrine, paracrine and endocrine activities. SASP proteins play a crucial role in influencing the behavior of neighboring cells in the surrounding microenvironment and induce paracrine senescence in normal human and mouse cells both in culture and *in vivo* (40). The SASP group includes a variety of cytokines, chemokines, growth factors, proteases and protease inhibitors. Specific proteins within this group are interleukins such as IL6, CXCL8 (IL8), cytokines such as TNF (TNFα) and CSF2 (GM-CSF), proteases (MMPs) and protease inhibitors (Serpins). Among the multiple SASPs components mediating paracrine senescence, a major role is played by Transforming Growth Factor-β family ligands.

A comprehensive survey of SASPs has been performed by Basisty et al. (41). These authors published a proteomic database of soluble exosomal factors released in the culture medium by different types of cells after stimulation by multiple senescence inducers (SASP atlas). Senescence was induced in the cultured cells by X-irradiation (IR), inducible RAS overexpression (RAS), or Atazanav treatment (a protease inhibitor used in HIV treatment) and allowed 1 to 2 weeks for the senescent phenotype to develop. Several proteins were secreted at much higher levels by treated cells compared with untreated, non-senescent cells. Each treatment resulted in the enhancement of more than hundred distinct proteins including a subset of proteins enhanced by all treatments (core SASPs). More recently the same group analyzed plasmas of 1,201 human subjects and identified the association of some of these SASP proteins with aging-related traits representing multiple aspects of physiology (42). In the performed meta-analysis, 28 of 77 SASPs proteins were significantly associated with age. Of these 28 age-associated SASPs proteins, IGFBP2 was associated with the most traits (8 traits), followed by GDF15 (7 traits), and CST3 (Cystatin-C) (5 traits). About 45% of the proteins that we found enhanced 1.2 folds or more in the old plasma group are included also in the above described SASP atlas. In our study, we observed an enhancement of Cystatin-C (2.5-folds) in old plasmas. IGFBP2 was included in the 612 identified proteins but not enhanced in the old plasmas whereas we have only partial information on GDF15 since the protein was enhanced in a semiquantitative microarray assay but was not among the 612 proteins identified by mass spectrometry. Other proteins enhanced in old plasmas in both our analysis and the analysis of Evans et al. (42) were Periostin (POSTN) and SERPING1.

A subgroup of SASPs is the Damage-Associated Molecular Patterns (DAMPs) released from necrotic cells or secreted from activated cells in response to tissue damage to trigger inflammation and immune responses in the attempt to eliminate pathogens and promote tissue repair. DAMPs become immunologically active upon tissue damage during both infectious and sterile insults. It has been reported that DAMPs are also implicated in the pathogenesis of cancer and autoimmune diseases, such as Rheumatoid Arthritis (RA) and atherosclerosis. DAMPs molecules released by damaged or dying cells serve as danger signals to alert the immune system and include, altered membrane phospholipids, extracellular proteins, such as mannose-rich glycans, and hyaluronan, nuclear protein such as heat shock proteins, S100 proteins, and histones. Other molecules present within cells, that leak out of damaged cells are ATP and nucleic acids. In our analysis, we found that several proteins classifiable as DAMPs were enhanced in the old plasmas. Old plasmas are also characterized by an increase of components of the complement activation and the coagulation cascades.

The enhanced components of complement are involved in the abnormal modulation of virtually all phases of an acute inflammatory reaction, that is changes in vascular flow and vessel diameter, an increased vessel permeability, extravasation and chemotaxis of leukocytes (43). The cells of the innate immune system and activated endothelial cells and platelets are involved in acute and chronic inflammation by releasing pro-inflammatory mediators, proteases and its inhibitors, clotting factors and associated proteins. Moreover, they expose adhesion molecules and specific receptors. There is a growing body of evidence that inflammation leads to stimulation of coagulation, and that in turn, coagulation activates inflammation (44). Vascular inflammation is associated with atherogenesis and increased thrombogenesis.

We hypothesize that physiologically aging cells and cells damaged by the sequel of micro and macro microbiological, traumatic, and dysmetabolic insults occurring during life release SASPs and DAMPs. The released antigens could evoke an immune-response and the formation of immune-complexes that activate the complement cascade and induce hemostasis and blood coagulation. Complement system and coagulation cascade proteins, particularly protease inhibitors such as Serpins, were reported as prominent plasma biomarkers of aging (21). This sequence of events leads to secretion of additional Amyloid Precursor Proteins and eventually results in a chronic inflammatory condition.

On top of this, a dramatic event such as the fracture occurring in our old orthopedic subgroup can evoke an acute phase response characterized by a rapid and exceptionally high increase in the plasma concentration of a restricted number of inflammatory proteins (see also the cluster formed by the last 10 proteins in **Figure 6** of this article).

In this article we identified 25 proteins enhanced in the plasmas of old individuals regardless of their life-experience and we propose these proteins as a signature suggestive of a chronic inflammatory state present in elderly. The protein list includes serum amyloid proteins recruiting immune cells to inflammatory sites and inducing enzymes that degrade extracellular matrix. The immune cells infiltration is facilitated by modification of the extracellular matrix catalyzed by coagulation factors and other present proteins such as PCOLCE. Members of the coagulation cascade can also increase vascular permeability and act as chemotactic agents for phagocytic cells. Other proteins are activators of signaling pathways. S100 protein, in particular, function as cytokine and bind with multi-ligand receptors of the immunoglobulin superfamily, that act as mediators in the pro-inflammatory signaling cascade, thus contributing to immune cell recruitment. Several proteins are members of the complement cascade. Some proteins fight microbial infection by reducing microbe growth through opsonization (CRP) or inhibition of iron uptake (HP) or entrapping invading microorganisms in blood clots. For some proteins the association with inflammation is well documented, but there is not a consensus on their role in the process. Interestingly some of these proteins could be inhibitors of inflammatory factors and play a role in a fine tuning of inflammation. One of these inhibitory proteins is SOD3 that inhibits the expression of pro-inflammatory cytokines, including TNF (TNFα), IL6, and CXCL8 (IL8). SOD3 is a member of the Superoxide Dismutase (SOD) protein family catalyzing the conversion of superoxide radicals into hydrogen peroxide and oxygen, to protect tissues from oxidative stress. The protein secreted into the extracellular environment is anchored to the extracellular matrix and cell surfaces via heparan sulphate proteoglycan and collagen. A fraction of the protein is proteolytically cleaved to generate circulating tetramers.

A study leading to the definition of a proteomic signature of aging was also performed by Tanaka et al. (21). However, these authors evaluated protein content in plasmas of human subjects of an uninterrupted age range (22 to 93 years) and only from individuals who were disease- and treatment-free and had no physical and cognitive impairment. They created a proteomic signature of chronological age based on relative concentrations of 76 proteins, Growth/Differentiation Factor 15 (GDF15) having the strongest, positive association with age. On the contrary, we investigated plasmas from a homogeneous age group of elderly people versus an age homogeneous young people group. Moreover, in the old group we separately considered subgroups of subjects with different life-experience. There were many overlaps between the proteins of the Tanaka et al. proteomic signature of aging and the proteins listed in **Table 2** of this article that we identified as enhanced in old plasmas. However, by our approach we were able to limit the signature to a much more restricted number of proteins. As already shown in **Figure 1**, the plasma protein content changes over the course of individual life (Old vs. Young) are strongly influenced by life-style and pathological conditions (O-ct vs. O-o and O-g).

We also investigated whereas significant differences exist regarding nature and concentration of proteins in male and female plasmas from old and young individuals as two separate groups. More than 40 proteins were significantly and differently expressed in young males and females. Some of these proteins have the capacity of binding or interacting with steroid hormones and could play a role in controlling the expression and the activity of sexual hormones. Similar comparisons were previously made by other authors, but they searched for sex-associated differences without distinguishing age groups. Different age groups at 10 year intervals were considered by Tanaka et al. who showed dependence of 8 genes from age and sex (21, 39). Other authors reported changes with age, with respect to fold change and statistical significance for proteins that differed with sex (24, 30). By utilizing the SomaScan^®^ platform in a sex-stratified analysis, Sathyan et al. identified 564 significant age-associated proteins in males compared to 274 proteins in females (45). However, a detailed comparison of sex differences was not performed by these authors. In a population of 240 healthy men and women, 22–93 years old, who were disease- and treatment-free and had no physical and cognitive impairment, eight proteins - Follicle-Stimulating Hormone (FSH), Sex Hormone-Binding Globulins (SHBG), Tissue Factor Pathway Inhibitor (TFPI), Luteinizing Hormone (CGA/LHB), Vitamin K-dependent protein 5 (PROS1), human Chorionic Gonadotropin (CGA/CGB), Netrin-4 (NTN4), and Insulin-like Growth Factor Binding Protein 7 (IGFBP7) - were differently associated by age and sex (21). Interestingly, the SHBG was the protein that in our analysis, after PZP, was the one with the highest enrichment (almost 4 folds) in the young female plasmas compared to young men plasmas. It is known that SHGB has different affinity for steroid hormones lowering their availability to the body (46). In an excellent article published in February 2025, Niu et al. by mass spectrometry analyzed plasmas from 2,147 children and adolescents and identified 1,216 proteins (47). Interestingly, several proteins reported as sex-modulated in children and adolescents in their study overlapped with those identified in our young cohort. Specifically, among the proteins upregulated in females, SHBG, PZP, FITM1, AGT, FGL1, and SERPINA7 were noted.

However, all published articles report significative differences between male and female plasmas. Therefore, it was unexpected our finding that no significative differences existed between protein content of plasmas from old males and females but a higher concentration of a single protein, the PZP, in the female plasmas. It is known that it inhibits the activity of several classes of proteinases. After its binding to a proteinase, the PZP conformation changes to trap the proteinase, limiting its activity. Moreover, PZP shows an immunosuppressive activity believed to suppress T-cell function during pregnancy to prevent fetal rejection. Because of its T-cell suppressing capability, it has been suggested also a possible role of PZP in regulation of tumor immune microenvironment (24).

In elderly plasmas we also observed a decreased production of antibody chains, especially kappa and lambda light chains. Together with IgM, kappa and lambda light chains are crucial for the first immune response. A reduced production of light chains in elderly people can result in a compromised adaptive immune response, a reduced antibody diversity, and a higher susceptibility to infections. These immune dysfunctions can also lead to re-activation of latent infections, decreased tumor immune-surveillance, and age-associated chronic immune-pathologies. In several cases the observed alterations involve also the antibody variable regions. During the whole life, IGKV and IGLV genes undergo continuous rearrangements to increase antibody diversity. However, with aging this capacity is progressively reduced. The coexistence of a reduced variability of the antibody repertoire due to the alterations in the light chain rearrangements and the chronic inflammation present in elderly people, defined “inflammaging” by some authors can further compromise an efficient immune response (23, 48).

## 5 Conclusions

Plasma protein profiling of 229 blood donors - including a prepubertal group, a healthy young adult group, and individuals over 75 years old with different life-experience - revealed a chronic inflammatory condition in the elderly. This included expression of immune response, complement activation and impaired regulation of blood coagulation. In addition, a reduced production of antibody light chains was observed in the older group, suggesting a concurrent immunological aging. We identified 25 proteins whose increased levels, alongside immunesenescence, may represent a plasma signature of aging. Notably, a life-experience that supports the maintenance of good physical and/or cognitive function appears to attenuate this aging-associated signature. No significant sex differences were observed in the plasma of elderly individuals, except for the increased presence of a single protein (PZP) in females. In contrast, several differences were identified when comparing plasma profiles of young males and females.

## Data Availability

The full sets of mass spectrometry proteomics data have been deposited in the ProteomeXchange Consortium via the PRIDE partner repository with identifier PXD063248. [Reviewer account details: Username: reviewer_pxd063248@ebi.ac.uk. Password: GmfWBwBk1cwH]. The other datasets used and/or analysed during the current study are available from the corresponding author on reasonable request.

## Supplementary material

Supplementary Table 1 SASPs present in at least one of the three groups: Old, Young and Children in the Old vs. Young and Old vs. Child comparisons.
