# Supplementary material for: Exploring the impact of age, sex and life experiences on plasma inflammatory profiles through comparative proteomics
Source: Front Immunol. 2026 Jan 7;16:1695213. doi: 10.3389/fimmu.2025.1695213 (PMC12819284; doi:10.3389/fimmu.2025.1695213)
Supplement: Supplementary Table 1 — SASPs present in at least one of the three groups: Old, Young and Children in the Old vs. Young and Old vs. Child comparisons. [file Table1.docx]

**Supplementary Table I SASPs present in at least one of the three groups: Old, Young and Children in the Old vs. Young and Old vs. Child comparisons.**

|  |  |  | **Compared to young** | | | **Compared to child** | | |
| --- | --- | --- | --- | --- | --- | --- | --- | --- |
| **Entrez Gene Symbol** | **UniProt number** | **Full name** | ***Fold increase*** | ***Significance*** | | ***Fold increase*** | ***Significance*** | |
|  |  |  |  | ***p_(-log10)_*** | ***q*** |  | ***p_(-log10)_*** | ***q*** |
|  |  |  |  |  | |  |  |  |
| P0DJI9 | SAA2 | Serum amyloid A-2 protein | 24.6 | 26.6 | 0.0001 | 15.7 | 6.6 | 0.0001 |
| P05109 | S100A8 | Protein S100-A8 | 3.3 | 17.8 | 0.0001 | -1.3 | 0.7 | 0.3998 |
| P61769 | B2M | Beta-2-microglobulin | 3.3 | 33.6 | 0.0001 | 4.3 | 14.6 | 0.0001 |
| P13647 | KRT5 | Keratin, type II cytoskeletal 5 | 3.3 | 3.9 | 0.0001 | 1.9 | 0.7 | 0.3019 |
| P08294 | SOD3 | Extracellular superoxide dismutase [Cu-Zn] | 3.0 | 15.2 | 0.0001 | 3.0 | 6.1 | 0.0009 |
| P06702 | S100A9 | Protein S100-A9 | 2.7 | 13.5 | 0.0001 | -1.4 | 0.8 | 0.3248 |
| Q12805 | EFEMP1 | EGF-containing fibulin-like extracellular matrix protein 1 | 2.7 | 13.3 | 0.0001 | 4.4 | 10.3 | 0.0001 |
| P01034 | CST3 | Cystatin-C | 2.5 | 12.4 | 0.0001 | 1.9 | 2.3 | 0.0565 |
| Q06033 | ITIH3 | Inter-alpha-trypsin inhibitor heavy chain H3 | 2.5 | 45.7 | 0.0001 | 2.2 | 11.4 | 0.0001 |
| Q15113 | PCOLCE | Procollagen C-endopeptidase enhancer 1 | 2.3 | 9.9 | 0.0001 | 2.4 | 3.4 | 0.0159 |
| P00738 | HP | Haptoglobin | 1.8 | 19.8 | 0.0001 | 2.3 | 8.7 | 0.0001 |
| Q99969 | RARRES2 | Retinoic acid receptor responder protein 2 | 1.7 | 6.5 | 0.0001 | 2.2 | 4.7 | 0.0062 |
| P14543 | NID1 | Nidogen-1 | 1.7 | 3.2 | 0.0017 | -1.3 | 0.4 | 0.5197 |
| Q6EMK4 | VASN | Vasorin | 1.7 | 6.2 | 0.0001 | 1.0 | 0.1 | 0.8815 |
| P33908 | MAN1A1 | Mannosyl-oligosaccharide 1,2-alpha-mannosidase IA | 1.6 | 19.1 | 0.0001 | 1.4 | 4.0 | 0.0558 |
| P13645 | KRT1 | Keratin, type I cytoskeletal 10 | 1.6 | 1.2 | 0.0363 | 1.1 | 0.1 | 0.8692 |
| P12259 | F5 | Coagulation factor V | 1.5 | 20.4 | 0.0001 | 1.4 | 5.5 | 0.0328 |
| P01031 | C5 | Complement C5 | 1.5 | 29.6 | 0.0001 | 1.6 | 12.6 | 0.0016 |
| P02533 | KRT14 | Keratin, type I cytoskeletal 14 | 1.5 | 0.6 | 0.1395 | 1.0 | 0.0 | 0.9561 |
| P19320 | VCAM1 | Vascular cell adhesion protein 1 | 1.5 | 6.4 | 0.0004 | 1.4 | 2.4 | 0.1206 |
| P10643 | C7 | Complement component C7 | 1.4 | 15.8 | 0.0001 | 1.1 | 1.1 | 0.4223 |
| O14786 | NRP1 | Neuropilin-1 | 1.4 | 5.0 | 0.0016 | 1.9 | 5.5 | 0.0063 |
| P23142 | FBLN1 | Fibulin-1 | 1.4 | 5.5 | 0.0018 | 1.4 | 3.0 | 0.1145 |
| O43447 | PPIH | Peptidyl-prolyl cis-trans isomerase H | 1.4 | 2.1 | 0.0148 | 2.3 | 4.0 | 0.0097 |
| P02743 | APCS | Serum amyloid P-component | 1.4 | 11.1 | 0.0002 | 2.0 | 15.4 | 0.0001 |
| P07195 | LDHB | L-lactate dehydrogenase B chain | 1.4 | 2.3 | 0.0131 | -1.4 | 1.5 | 0.1774 |
| Q15063 | POSTN | Periostin | 1.3 | 1.4 | 0.0400 | -2.5 | 2.9 | 0.0253 |
| P14625 | HSP90B1 | Endoplasmin | 1.3 | 4.3 | 0.0036 | 1.3 | 1.7 | 0.1896 |
| P25311 | AZGP1 | Zinc-alpha-2-glycoprotein | 1.3 | 8.8 | 0.0008 | 1.7 | 9.3 | 0.0016 |
| P05155 | SERPING1 | Plasma protease C1 inhibitor | 1.3 | 14.5 | 0.0004 | 1.1 | 1.1 | 0.4641 |
| P20742 | PZP | Pregnancy zone protein | 1.3 | 0.7 | 0.1291 | 1.6 | 1.1 | 0.2000 |
| P02671 | FGA | Fibrinogen alpha chain | 1.3 | 12.7 | 0.0008 | 1.6 | 10.9 | 0.0018 |
| Q13740 | ALCAM | CD166 antigen | 1.3 | 1.6 | 0.0420 | 1.1 | 0.2 | 0.7871 |
| P16070 | CD44 | CD44 antigen | 1.3 | 1.4 | 0.0560 | -1.1 | 0.3 | 0.6604 |
| O00391 | QSOX1 | Sulfhydryl oxidase 1 | 1.3 | 11.9 | 0.0016 | 1.2 | 2.8 | 0.1850 |
| P24592 | IGFBP6 | Insulin-like growth factor-binding protein 6 | 1.3 | 3.7 | 0.0130 | 2.9 | 14.0 | 0.0001 |
| P07225 | PROS1 | Vitamin K-dependent protein S | 1.3 | 12.2 | 0.0020 | 1.1 | 1.5 | 0.4089 |
| P49747 | COMP | Cartilage oligomeric matrix protein | 1.2 | 1.4 | 0.0620 | -1.5 | 1.3 | 0.1930 |
| Q92820 | GGH | Gamma-glutamyl hydrolase | 1.2 | 1.3 | 0.0666 | 1.2 | 0.6 | 0.4616 |
| P27797 | CALR | Calreticulin | 1.2 | 1.1 | 0.0892 | 1.3 | 0.8 | 0.3664 |
| P13645 | KRT10 | Keratin, type I cytoskeletal 10 | 1.2 | 0.4 | 0.2976 | 1.1 | 0.1 | 0.9238 |
| P05156 | CFI | Complement factor I | 1.2 | 15.2 | 0.0036 | 1.1 | 2.0 | 0.3784 |
| P0C0L4 | C4A | Complement C4-A | 1.2 | 2.7 | 0.0415 | 1.3 | 1.6 | 0.2163 |
| Q04756 | HGFAC | Hepatocyte growth factor activator | 1.2 | 4.4 | 0.0263 | 1.2 | 2.4 | 0.1774 |
| P02538 | KRT6A | Keratin, type II cytoskeletal 6A | 1.2 | 0.5 | 0.2654 | 1.5 | 0.8 | 0.3169 |
| P02675 | FGB | Fibrinogen beta chain | 1.2 | 6.1 | 0.0211 | 1.3 | 5.3 | 0.0603 |
| Q9NZP8 | C1R | Complement C1r subcomponent-like protein | 1.2 | 9.5 | 0.0228 | 1.3 | 6.5 | 0.0732 |
